# Fetal Alcohol Spectrum Disorder: Potential Role of Endocannabinoids Signaling

**DOI:** 10.3390/brainsci5040456

**Published:** 2015-10-29

**Authors:** Balapal S. Basavarajappa

**Affiliations:** 1 Division of Analytical Psychopharmacology, Nathan Kline Institute for Psychiatric Research, Orangeburg, NY 10962, USA; 2 New York State Psychiatric Institute, College of Physicians & Surgeons, Columbia University, New York, NY 10032, USA; 3 Department of Psychiatry, College of Physicians & Surgeons, Columbia University, New York, NY 10032, USA

**Keywords:** fetal alcohol, learning and memory, CB1 receptors, brain development, synaptic plasticity, intellectual disability

## Abstract

One of the unique features of prenatal alcohol exposure in humans is impaired cognitive and behavioral function resulting from damage to the central nervous system (CNS), which leads to a spectrum of impairments referred to as fetal alcohol spectrum disorder (FASD). Human FASD phenotypes can be reproduced in the rodent CNS following prenatal ethanol exposure. Several mechanisms are expected to contribute to the detrimental effects of prenatal alcohol exposure on the developing fetus, particularly in the developing CNS. These mechanisms may act simultaneously or consecutively and differ among a variety of cell types at specific developmental stages in particular brain regions. Studies have identified numerous potential mechanisms through which alcohol can act on the fetus. Among these mechanisms are increased oxidative stress, mitochondrial damage, interference with the activity of growth factors, glia cells, cell adhesion molecules, gene expression during CNS development and impaired function of signaling molecules involved in neuronal communication and circuit formation. These alcohol-induced deficits result in long-lasting abnormalities in neuronal plasticity and learning and memory and can explain many of the neurobehavioral abnormalities found in FASD. In this review, the author discusses the mechanisms that are associated with FASD and provides a current status on the endocannabinoid system in the development of FASD.

## 1. Introduction

The influence of alcohol use during pregnancy on children has received widespread research attention since early reports that prenatal alcohol exposure can have devastating and enduring consequences [1]. Among the many potential outcomes of prenatal alcohol exposure, alterations in the developing brain and the resulting lifelong neurobehavioral deficits have been the most devastating. One of the many recognized consequences of prenatal alcohol exposure is fetal alcohol syndrome (FAS) [2], which is characterized by pre- and postnatal growth deficiencies, craniofacial anomalies and central nervous system (CNS) dysfunction. FAS is now recognized as an important cause of intellectual disabilities and behavior problems in many countries [3,4,5,6,7]. However, over the last 35 years, it has become clear that FAS is not the only outcome resulting from prenatal alcohol exposure. Indeed, the term fetal alcohol spectrum disorders (FASD) [8] has been used to emphasize the continuous nature of the effects of prenatal alcohol exposure, with FAS at the more severe end of the spectrum. While literature regarding the behavioral profile of FASD is relatively limited [9], reported deficits suggest that FASD is one of the main causes of intellectual disability in Western nations [10] and is accompanied by widespread neuropsychological deficits such as verbal learning/recall abilities [11,12], including deficits in learning and memory [13,14]. These substantial neuropsychological deficits create many daily challenges for individuals with FASD. However, the cognitive deficits of FASD are not fully understood, and a characteristic outline has not been identified. This review will provide a comprehensive summary of the neurobehavioral effects of heavy prenatal alcohol exposure. Supporting studies from animal model systems with an emphasis on the molecular events mediated by the endocannabinoid system resulting in brain-behavior associations are included when appropriate.

### 1.1. Influence of Developmental Stage, Amount and Pattern of Alcohol Use on Neurobehavioral Outcome

The consequences of maternal alcohol use during pregnancy on the neurobehavioral outcome of children are dependent on several factors, including the amount and pattern of alcohol consumption as well as the developmental timing of exposure. In most cases, the amount of alcohol consumed is correlated with the severity of the outcome [15,16]. However, the pattern of alcohol exposure can often undermine these effects, with binge-like exposures resulting in more severe deficits than chronic exposure [17,18]. Developmental timing of the exposure is also important. Alcohol exposure at different periods of fetal development can greatly influence the pattern and severity of the structural and functional abnormalities [19]. In addition, several other factors may contribute to this variation in the consequences of maternal drinking. These factors include, but are not limited to, the following: differences in maternal metabolism, differences in genetic susceptibility, and variation in the vulnerability of different brain regions. Nevertheless, factors that alter the peak blood alcohol concentration (BAC) experienced by the embryo or the fetus (e.g., maternal drinking patterns and maternal metabolism) are most likely to affect the occurrence and severity of alcohol-induced developmental brain disorders. Unfortunately, this level of detail is often difficult to document, particularly in retrospectively recruited samples, and individual studies provide varying degrees of detail concerning the levels and patterns of exposure. These issues highlight the modifying variables that contribute to the considerable range of phenotypes presented in children with FASD.

### 1.2. Neurobehavioral Abnormalities in Children with FASD

FASD affects an estimated prevalence of as high as 2%–5% in the United States and several Western European countries [20] and is the leading preventable cause of mental retardation [8]. Although publicity campaigns have encouraged women to stop alcohol consumption once they know that they are pregnant, data from a US national survey indicated that 13% of women continue to use alcohol during pregnancy (see [21]).

Prenatal alcohol exposure has been associated with widespread neuropsychological deficits across several domains (Table 1), including general intelligence, memory, language, attention, learning, visuospatial abilities, executive functioning, fine and gross motor skills and social and adaptive functioning [12]. Many FASD individuals exhibit impaired intellectual abilities and growth retardation [22], although children with a diagnosis of FAS tend to have more severe impairments compared to those who were exposed prenatally to alcohol but do not have sufficient dysmorphic features for a diagnosis [23]. However, not all individuals diagnosed with FAS are intellectually disabled (IQ score < 70), and intellectual disability is not a necessary criterion for the diagnosis of FAS. The average IQ estimate of children with prenatal alcohol exposure is 70 for those with FAS [7] and 80 for nondysmorphic children [23]. However, fewer studies have been performed on the intellectual abilities among individuals with moderate levels of alcohol exposure, and these results have been inconsistent [16,24,25,26,27]. The discrepancies among studies with moderate levels of exposure warrant the need for further research and the importance of considering a variety of factors as described in the proceeding section when evaluating studies.

**Table 1:** Summary of neuropsychological abnormalities found in individuals with fetal alcohol spectrum disorders (FASD) when compared to typically developing children.

Cognitive Function	FASD	References
General Intelligence	Lower IQ (~70)	[7,23,28]
Executive Function	Impaired executive functions such as planning, fluency and working memory	[14,29,30,31,32,33,34]
Learning and memory		
Verbal	Impaired initial learning without affecting retention of information already learned due to implicit learning strategies.	[12,35,36,37,38,39,40]
Nonverbal	Impaired nonverbal learning and memory but impairment of retention of information is inconsistent.	[29,36,39,41,42,43,44]
Motor function	Deficits in motor abilities and visual-motor tasks.	[11,35,42,45,46,47,48,49,50,51]
Attention and hyperactivity	Deficits in attention and exhibit hyperactivity.	[10,48,52,53,54,55,56,57,58,59,60]

A number of clinical studies have also reported learning and memory deficits in children with heavy prenatal alcohol exposure. Prenatal alcohol-exposed children display poorer academic achievement and higher learning disability rates than control children [61], which may relate to impairments in their verbal and non-verbal learning and memory [35,36,37]. These deficits were present in both children with and without the dysmorphic features of FAS [12,36]. Some studies have suggested that the long-term retention of verbal information is intact in alcohol-exposed children but that the initial encoding processes may be impaired [36,38]. In addition to non-verbal memory difficulties, children with FASD showed visuospatial processing deficits [35], suggesting damage and abnormalities in the frontal-subcortical pathway. Attention deficits [62,63] and attention deficit hyperactivity disorder (ADHD) [64] were also frequently found in FASD children. A recent collaborative study also suggested that executive function and spatial processing were particularly sensitive to prenatal alcohol exposure [52]. Executive functions are regulated by the frontal-subcortical circuits, which involve projections from the frontal lobes to the basal ganglia and thalamic nuclei [65]. These regions have been found to be affected by prenatal alcohol exposure [66,67]. In addition, FASD children have social and adaptive deficits because they are more likely to be rated as hyperactive, disruptive, impulsive or delinquent compared to non-exposed children [68,69]. Furthermore, children with prenatal alcohol exposure have difficulty resolving conflicts and in anticipating the consequences of their actions [70,71], suggesting the challenges that children with FASD face in interacting with peers.

In summary, the phenotype of children with FASD is characterized by social, behavioral, and language problems that disrupt interactions with peers and appear to be influenced by task complexity. In addition to these disabilities, neuropsychological problems in planning, inhibition, self-regulation, and attention and memory potentially contribute to the challenges faced by these children in school settings. In particular, children with FASD may suffer from social and academic situations because their deficits interfere with their ability to be sufficiently engaged. Student engagement, inferred from performance (attention, work completed, appropriate participation), is key to motivation and, ultimately, learning or mastering knowledge or skills [72]. These observations highlight not only the importance in identifying FASD at early ages, but also the provision of specific treatments to address this developmental disability because early identification and treatment have been demonstrated to be protective against more serious secondary disabilities [73,74,75]. Despite four decades of progress, research on FASD and its treatment continue to be hampered by a lack of specificity in the behavioral diagnostic criteria and a limited understanding of the cellular and molecular substrates of cognitive deficits (e.g., attention, memory, executive functioning) caused by heavy prenatal alcohol consumption. Finally, research on the biomarkers of fetal alcohol damage may help to improve the future of children affected by prenatal alcohol exposure.

### 1.3. Neurobehavioral Abnormalities in Animal Models of FASD

Animal models provide rigorous experimental control over numerous potentially confounding variables as well as control of the dose and timing of the alcohol exposure. In addition, animal models have the advantage of non-confounding multiple factors, which may be independently manipulated. In contrast, causal conclusions are difficult to reach in human studies due to the clustering of negative events. For example, alcohol consumption and smoking tobacco are often strongly associated with each other, and alcohol use usually affects nutrition intake in people. However, to relate animal models to human development, the relative states of brain development at times of alcohol exposure need to be carefully considered. Some of the behavioral and anatomical outcomes discussed above have also been reported in animal models of FASD. A number of researchers have extended the findings in humans to animal research using tests that have been found to be sensitive to hippocampal function [41,76,77,78,79,80,81,82,83,84,85,86].

Prenatal alcohol exposure has been shown to affect embryonic development, which varies depending on the severity, duration, and frequency of exposure of ethanol during gestation [87,88,89]. During this period, the use of alcohol prevents the proper blastocyst implantation in the uterus, resulting in an increased rate of resorption or early termination of the pregnancy, which generally occurs before a woman realizes she is pregnant. In the third week after fertilization, specific alcohol-induced birth defects begin to affect the developing embryo [90,91,92,93]. Between the third and sixth week after fertilization, when neurulation occurs, the cranial neural crest cell population is vulnerable to alcohol-induced damages [94], which are related to facial abnormalities characteristic of FAS. From the third week through the third trimester, the cellular progenitor pools, known as radial glia, which will give rise to the CNS, become vulnerable to the effects of alcohol and result in morphological abnormalities and an overall reduction in white matter within the brain [95,96]. Specific damage to the brain continues in the sixth and seventh week following fertilization, after the brain has begun to divide into vesicles. The corpus callosum, a midline structure responsible for communication between the left and right hemispheres of the brain, has been shown to be vulnerable to alcohol at this stage of development [97]. The eighth week after fertilization is the end of the embryonic stage and the beginning of the fetal stage of pregnancy. At this stage, the developing brain remains vulnerable to prenatal exposure to alcohol, particularly in the formation of the cerebellum, and the fetus remains vulnerable in terms of prenatal growth restrictions. The cerebellum and hippocampus are vulnerable to the neurotoxic effects of alcohol [98], particularly when alcohol exposure is limited to the third trimester equivalent in the rat (*i.e.*, the first postnatal week) [98,99]. Purkinje cells within the cerebellum [100] appear most vulnerable to the effects of alcohol prior to the 7th postnatal day (PD) [101], while alcohol exposure encompassing all of gestation (gestational days (GD)1–22) or limited to either PD 4–9 or PD 7–9 results in CA1 pyramidal cell reduction within the hippocampus [80,82,84,102,103,104,105]. Limiting alcohol exposure to PD 7–9, therefore, is likely to result in significant hippocampal targeting relative to the cerebellum and may result in severe learning and memory deficits [106,107,108,109,110,111,112].

#### 1.3.1. Potential Mechanisms Responsible for FASD

Anatomical alterations associated with brain dysfunction may range from a gross reduction in brain volume to deficits in cell number in a particular brain region to cellular modifications of individual neurons, resulting in alterations in the communications among neuronal cells. These alterations can have a long-term detrimental effect on behavioral and cognitive development. Experimental evidence has demonstrated that ethanol exposure during brain development could affect several molecular, biochemical and cellular events, which participate in the proper formation of brain structure. Thus, at the cellular level, and depending on the time and amount of alcohol exposure, ethanol could induce deficits in cell proliferation, migration, growth and differentiation and may cause apoptotic neurodegeneration. For example, alcohol exposure during early embryonic development, when cells undergo rapid division, causes a reduction in the number of cells generated [113], including neural progenitor cells [114,115,116], abnormal neuronal migration and may cause subsequent cell death [117,118]. However, if alcohol exposure occurs during the cell differentiation [119,120] and synaptogenesis period [121], it may cause robust neurodegeneration [111,112,121,122,123,124] leading to the loss of synapses. These results support the notion that ethanol exerts its effects by multiple actions at different sites involving several mechanisms.

Several major molecular mechanisms have been identified as potential candidates responsible for FASD [98,125,126]. These mechanisms are likely to participate at different stages of brain development and/or with different doses of ethanol. The major probable mechanisms include the following: (1) alterations in the regulation of gene expression (e.g., expression of anandamide (AEA) and 2-arachidonylglycerol (2-AG) synthesizing enzymes, 2-AG metabolizing enzyme, cannabinoid receptor type 1 [111,127], homeobox gene, histone methyl transferase, altered histone and DNA methylation [109,110,124,128,129,130,131,132], and microRNAs (miRNAs) [133,134]; (2) interference with mitogenic and growth factor responses involved in neural stem cell proliferation, migration and differentiation [135,136,137]; (3) disturbances in the molecules that regulate cell–cell interactions (e.g., L1, NCAM, growth factors) [138,139,140,141,142,143]; (4) activation of the molecular cascade, which regulates cell survival or cell death (growth factor deprivation, oxidative stress, apoptotic signaling through caspase-3 activation, H3 histone methylation mediated histone degradation, suppression of *N*-Methyl-d-aspartate (NMDA), glutamate and gamma-amino butyric acid-A (GABAA receptors) [111,124,144,145,146,147,148,149,150]; (5) abnormalities in glial proliferation, differentiation and function [95,151] and (6) suppression of the mitogen-activated protein kinase (MAPK) pathway via enhanced endocannabinoid signaling [110,111,127]. This last mechanism is supported by CB1 receptor antagonist and CB1 receptor null mice studies revealing the contribution of this signaling system to neuronal loss through caspase-3 and NMDA receptors, synaptic plasticity and learning and memory abnormalities found in an FASD animal model [110,111,127]. Due to the potential contribution of this novel mechanism to brain development, learning and memory loss with FASD, we will describe the evidence demonstrating that the endocannabinoid signaling system is an important potential target involved in the development of FASD.

#### 1.3.2. Role of the Endocannabinoid System during Brain Development and in Ethanol-Induced Brain Abnormalities

Within the last two decades, cannabinoid research has received tremendous attention due to the breakthrough and discovery of the receptors that bind delta-9-tetrahydrocannabinol (Δ^9^-THC) (cannabinoid receptors) and their endogenous ligands, endocannabinoids (ECs) (endocannabinoid system) in animal tissues (for a recent review see [152]). This emerging body of research has revealed multiple ways in which the EC system functions to regulate synaptic neurotransmission in various brain areas [153,154,155] in the developing and adult brain. Growing research has demonstrated vital functions for EC signaling in molecular pathways that underlie both short- and long-lasting alterations in synaptic strength [156,157]. In fact, the critical involvement of ECs in some mechanisms of synaptic neurotransmission may change the current working cellular models of learning and memory. These models may be vital in understanding and providing potential treatment for the disease conditions in which the EC system plays an important role.

##### Potential Role of Endocannabinoids and Their Metabolic Enzymes

ECs consist of lipid signaling molecules that bind to and activate cannabinoid receptors. These lipid compounds are formed from phospholipid precursors [158,159,160,161,162,163] within cells throughout the body and are released from these cells on demand in a nonvascular manner to act in a paracrine fashion [158,160,161,162,163,164].

In 1992, anandamide (arachidonylethanolamide, AEA) was first identified as an endogenous cannabinoid. Its name is derived from the Sanskrit word, *ananda*, which means “internal bliss”, making a reference to its chemical structure (the amide of arachidonic acid and ethanolamine) [165]. Subsequently, another endogenous cannabinoid receptor ligand, 2-arachidonylglycerol (2-AG), was discovered and characterized [166,167]. The third ether-type EC, 2-arachidonylglycerol ether (noladin ether), was isolated from the CNS and shown to display pharmacological properties similar to AEA [168]. The fourth type of EC, virodhamine, in contrast to the previously described ECs, is a partial agonist with *in vivo* antagonist activity on the CB1 receptor [169]. The fifth type of EC, *N*-arachidonyl-dopamine (NADA), not only binds to the CB1 receptor but also stimulates vanilloid receptors (VR1) [170]. Importantly, except for AEA and 2-AG, to date, there is little evidence regarding the physiological actions of these compounds.

**Figure 1:**
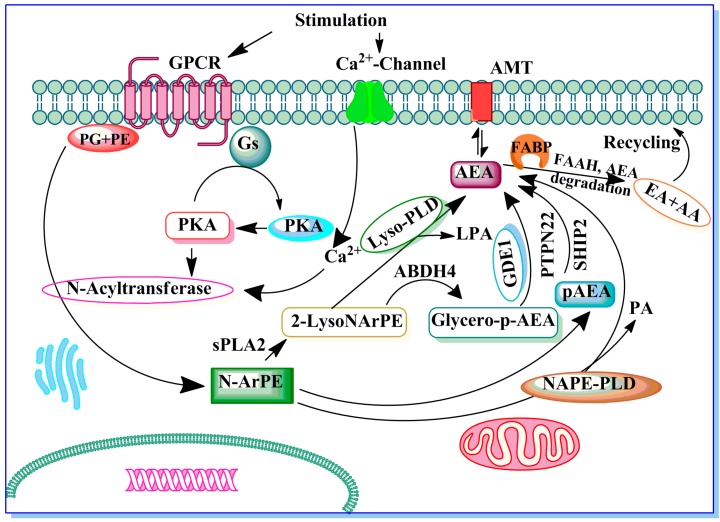
The potential enzymes involved in anandamide (AEA) biosynthesis. Stimulation of G-protein coupled receptor (GPCR) mediated adenylate cyclase and cAMP-dependent protein kinase (protein kinase A, PKA) potentiate *N*-acyltransferase (Ca^2+^-dependent transacylase) (NAT). A fatty arachidonic acid chain is transferred by NAT from the sn-1 position of phospholipids (phosphoglycerides, PG) to the primary amine of phosphatidylethanolamine (PE) in a Ca^2+^-dependent manner, forming an *N*-arachidonyl phosphatidylethanolamine (N-ArPE). This N-ArPE intermediate is then hydrolyzed by a phospholipase D (PLD)-like enzyme to generate AEA. In addition, potential alternative pathways for AEA formation from N-ArPE through 2-lysoNArPE and pAEA catalysed by sPLA2, lyso-PLD, ABDH4, GDE1 or PTPN22 and SHIP2 are shown (see text for details). Once synthesized, AEA can be metabolized into ethanolamine (EA) and arachidonic acid (AA) or transported to the outside of the cell through a process that has not yet been well characterized. Fatty acid binding proteins (FABPs) serve as intracellular carrier of AEA and play an important role in the AEA degradation by FAAH. EMT, ECmembrane transporter.

AEA is thought to be synthesized by several pathways (see review for details [171]). Importantly, there is strong evidence for a calcium dependence in both of these synthesis steps, which may underlie the requirement for postsynaptic Ca^2+^ in specific forms of depolarization-induced synaptic plasticity (For details see [157]). Biosynthesis of AEA from membrane phospholipid precursors is catalyzed by several enzymes. The most well-studied of these is *N*-acylphosphatidylethanolamine-specific phospholipase D (NAPE-PLD; [172,173]), but others include glycerophosphodiesterase (GDE1) [174], abhydrolase domain 4 (ABHD4) [175] and phosphatase PTPN22 [176] (Figure 1). In our recent study, we found that NAPE-PLD protein levels were significantly lower during the synaptogenic period of mouse brain development and gradually increased to adult levels [111]. Similar patterns of NAPE-PLD expression were also found during rat brain development [177]. Overwhelming data suggested that the lack of NAPE-PLD significantly reduced the levels of AEA and other NAE [178,179] in the brain, although some controversy exists on whether NAPE-PLD contributes to the formation of AEA in the brain [180]. Interestingly, studies have suggested a significant reduction in the accumulation of brain NAEs, including AEA, in GDE1/NAPE-PLD double KO mice treated with an FAAH inhibitor that blocks NAE degradation [181]. Our study suggested that postnatal ethanol treatment enhanced AEA levels via transcriptional activation of both the NAPE-PLD and GDE1 genes, resulting in the enhanced expression of NAPE-PLD and GDE1 mRNA and proteins in neonatal mouse brain regions [111].

As a putative neuromodulator, AEA released into the synaptic cleft is expected to be rapidly inactivated. In general, two mechanisms are known that could remove ECs from the synaptic cleft to ensure rapid signal inactivation: re-uptake or enzymatic degradation. AEA is inactivated by reuptake [182,183] via an uncharacterized membrane transport molecule, the “AEA membrane transporter” (AMT) [163,182,184,185,186,187,188], and subsequent intracellular enzymatic degradation. AEA is metabolized into arachidonic acid and ethanolamine via the action of the fatty acid amide hydrolase (FAAH), and this activity plays a significant role in the rapid clearance of AEA from extracellular compartments [189,190]. In addition, fatty acid binding proteins (FABPs) are suggested to serve as intracellular carrier of AEA and play an important role in the pathway for AEA inactivation by FAAH. Inhibition of FAAH activity by pharmacological blockade or genetic ablation has been shown to cause an accumulation of AEA in the brain [191,192,193]. Similar to FAAH inhibition, inhibitors of FABPs also reduce hydrolysis of AEA and its uptake into cells, raising levels of extracellular AEA [194,195,196,197]. In addition to hydrolysis by FAAH, AEA is metabolized by COX-2, LOX and cytochrome P450 [187,198,199,200]. Further research is required to elucidate the precise mechanism and enzymes involved in this pathway of AEA metabolism. It was previously shown that the FAAH distribution during postnatal development was very similar to the adult pattern [201]. In our study, postnatal ethanol treatment did not considerably alter either the FAAH protein or the mRNA levels in neonatal brain regions [111]. Moreover, FAAH has been detected in radial glia during late gestation and postnatal periods [202]. These distribution patterns of FAAH, together with the EC control of astrogliogenesis [202], suggest the involvement of EC signaling in neural progenitor differentiation *in vivo*. A fine balance between progenitor cell proliferation and programmed death ensures the generation of adequate quantities of neural cells during brain development.

The second widely recognized endogenous CB1 agonist is 2-AG, which was characterized soon after the discovery of AEA [166,167]. 2-AG has been characterized as a unique molecular species of monoacylglycerol isolated from both the canine gut [166] and rat brain [203], where it presumably functions as an endogenous cannabinoid receptor ligand. 2-AG biosynthesis occurs via two possible routes in neurons (see recent review [204]). DAGL-α and β both contribute to a large extent the regulation of steady-state levels of 2-AG in the brain and other tissues. However in mice lacking DAGL-α, 2-AG levels are reduced by up to 80% in the brain and up to 50% in the DAGL-β null mouse brain [205]. DAGL-α and β are expressed lower during early development and gradually increase throughout development to adulthood [127]. 2-AG, similar to AEA, is found in a variety of tissues throughout the body and brain and appears to be released from cells in response to specific stimuli. Although the 2-AG concentration in the rodent brain is in the nanomolar range, which is one hundred times higher than that of AEA, it is 20 times less potent than AEA at the CB1 receptor [166]. 2-AG is also inactivated by reuptake [182,183] via an uncharacterized AMT [163,182,184,185,186,187,188], and subsequent intracellular enzymatic degradation [158,189,206] by monoacylglycerol (MAGL) lipase, similar to other monoacylglycerols [207]. MAGL expression levels are lower during early development and gradually increase throughout development to adulthood [127]. Furthermore, 2-AG is metabolized by enzymatic oxygenation of 2-AG by COX-2 into PGH2 glycerol esters. The biological activity and role of oxygenated 2-AG have yet to be determined. Furthermore, postnatal ethanol treatment did not significantly enhanced 2-AG levels in neonatal brain regions. Taken together, these findings raise an important question of whether AEA and 2-AG may exhibit distinct functions and warrant future studies to understand their specific role during brain development. In our recent studies postnatal ethanol treatment consistently enhanced DAGL-β and MAGL, resulting in no significant change in the 2-AG levels [127]. Although these events are not economical to cells’ overall function, these observations suggest that the enhancement of AEA over 2-AG by postnatal ethanol constitutes an example of the generation of unique cellular responses depending on the environment of the cell.

The molecular distribution of EC metabolism and their receptor systems during brain development suggest that ECs may effectively regulate cellular specification programs [208]. A broad range of developmentally regulated receptors and ion channels [209,210,211] has suggested divergent roles of EC signaling during brain development. AEA and 2-AG levels vary substantially throughout prenatal development [212,213]. In the beginning stages, between days four and six of pregnancy, AEA in the uterus enables embryo implantation [214]. AEA levels are low in the brain at midgestation, and their levels gradually increase throughout the perinatal developmental period until adult levels are reached [212]. Similar to adult brains, 2-AG concentrations (2–8 nmol/g tissue) exceed those of AEA (3–6 pmol/g tissue) throughout brain development [212,213]. Importantly, fetal 2-AG levels are similar to those in young and adult rat brains, with a remarkably distinct peak on the first day after birth [212,213]. It has become increasingly evident that ECs and related lipid mediators regulate neural progenitor commitment, survival [202,215,216] and synaptic connectivity in the developing brain [217,218].

ECs have also been shown to regulate neuronal migration, suggesting a role in specifying the morphological, physiological and molecular characteristics that occur during terminal neuronal differentiation. AEA and WIN552122 and cooperatively with brain-derived neurotrophic factor (BDNF), a principal pro-differentiating neurotrophin, induce migration of GABA-expressing interneurons in the embryonic cortex [219]. Similarly, Δ^9^-THC increased the density of cholecystokinin-expressing interneurons in the rat hippocampus *in vivo* [219]. AEA [219] and WIN552122 [220] strongly inhibited neurite formation and elongation in GABA-expressing interneurons. In these studies, AEA abolished the morphogenic potential of BDNF. Similarly, cannabinoids, including Δ^9^-THC, antagonized forskolin-induced synaptogenesis in cultured hippocampal neurons [221]. In N1E-115 neuroblastoma cells, AEA and HU210 reduced the rates of neurogenic differentiation [222]. These morphological changes were mediated through the Rho family of small guanosine triphosphatases and spatially controlled its activation, which regulated cytoskeletal integrity [223]. In contrast, the synthetic cannabinoid, HU210 promoted neurite outgrowth in Neuro 2A cells via Gαo/i-mediated degradation of Rap–GAPII and the subsequent activation of Rap1 [224]. 2-AG also stimulated neurite outgrowth in cerebellar neurons through a mechanism that was dependent on intrinsic diacylglycerollipase (DAGL) activity within axonal growth cones, whereas CB1 receptor antagonists abolished *N*-cadherin- and Fgf8-induced neurite extension [225]. These observations indicated that EC signaling might regulate aspects of growth cone differentiation and axon guidance [226]. Further support for the potential role of ECs in the regulation of neuritogenesis was obtained from the similar functional effects of other lipid mediators such as lysophosphatidic acid and sphingosine-1-phosphate [222]. Further research is required to understand the precise molecular events by which ECs regulate dendritic and axonal development. Identification of ECs and the characterization of their metabolic enzymes and second messenger signaling cascades will enable a better understanding of the physiological role of EC signaling and will reveal the neural basis of developmental defects that are imposed by prenatal drug abuse including ethanol.

##### A Major Role of Cannabinoid Receptors and Their Signaling

Evidence for the existence of a marijuana receptor has been demonstrated since the 1980s [227,228]. It has now been shown that cannabinoids consist of two specific receptor subtypes, known as CB1 and CB2, which have been cloned. Evidence for a third receptor (“CB3” or the “AEA receptor”) in brain and endothelial tissues has been reported in the literature [229,230,231,232]. However, the cloning, expression and characterization of CB3 has not yet been performed.

CB1 and CB2 receptors belong to a large superfamily of heptahelical G protein-coupled receptors (GPCR) and couple to Gi/o proteins (for more details, see reviews [171,233,234]). The CB1 receptor is mainly expressed in the brain and spinal cord and is thus often referred to as the “brain cannabinoid receptor”. CB1 receptors are among the most abundant GPCRs in the brain, and their expression levels are similar to the levels of GABA- and glutamate-gated ion channels [235]. The presence of functional CB2 receptors in the CNS has provoked considerable controversy over the past few years. Formerly considered as an exclusively peripheral receptor [236,237] and often referred to as a “peripheral cannabinoid receptor”, it is now accepted that CB2 is also present in limited amounts and in distinct locations in the brain of several animal species, including humans [238,239]. However, the functional relevance of this receptor in the CNS is emerging slowly [240].

The CB1 receptor displays a wide expression pattern in the developing nervous system and its expression corresponds with neuronal differentiation in the embryo from its earliest stages. Several studies have described the expression pattern of CB1 receptor mRNA and the distribution of CB1 receptors in the fetal and neonatal rat brain [212,241,242,243]. CB1 receptor mRNA levels and receptor binding can be detected from GD 14 in rats, which coincides with the time of phenotypic expression of most neurotransmitters (for review, see [244]). At this fetal age, CB1 receptors appear to be functional because they are already coupled to GTP-binding proteins [241]. The developing human and rat brain contain higher levels of CB1 receptors [245,246] compared to the adult brain [212]. However, the distribution of CB1 receptors is atypical in the fetal and early neonatal brain, particularly in white matter areas [243] and in the subventricular zones of the forebrain [212,241] compared to the adult brain [235,246]. This atypical location of CB1 receptors is a transient phenomenon because the receptors are progressively acquired during the course of late postnatal development, subsequently resulting in the classic pattern of distribution observed in the adult brain [241,243]. The existence of CB1 receptors during early brain development suggests the potential involvement of CB1 receptors during fetal and early postnatal periods in specific events of CNS development, such as cell proliferation and migration, axonal elongation and, subsequently, synaptogenesis and myelinogenesis (for review, see [213]). Thus CB1 receptors contribute to the generation of neuronal diversity in specific brain regions during early brain development.

Activation of a cannabinoid receptor promotes its interaction with G proteins, resulting in guanosine diphosphate/guanosine triphosphate exchange and subsequent dissociation of the α and βγ subunits. These subunits regulate the activity of multiple effector proteins to elicit biological functions. CB1 receptor is coupled with G_i_ or G_o_ proteins. CB1 receptors differ from many other GPCR proteins in that they are constitutively active because they are pre-coupled with G-proteins in the absence of exogenously added agonists [247]. Among its cellular actions is the inhibition of adenylate cyclase activity [248,249,250], inhibition of N- type voltage-gated channels [251,252,253,254], inhibition of N-type, P/Q-type calcium channels and D-type potassium channels [250,255], activation of A-type and inwardly rectifying potassium channels [256] and inhibition of synaptic transmission [255,257]. On the basis of these findings, it has been suggested that CB1 receptors play a role in the regulation of neurotransmitter release [255,257].

In addition, one of the most interesting research areas is the regulation of neuritogenesis, axonal growth and synaptogenesis by CB1 receptors (for references, see recent article [218]). However, the molecular mechanisms involved in this process are still unclear. The CB1 receptor activates the MAPK pathway [258]. In some cells, CB1 receptor-mediated activation of MAPK was mediated via the PI3 kinase pathway [258,259]. AEA, CP, 55,940 and WIN 55,212-2 increased phosphorylation of focal adhesion kinase (FAK^+^6,7) a neural isoform of FAK, in hippocampal slices and in cultured neurons [260]. Moreover, CB1 receptor activation stimulated the phosphorylation of the Tyr-397 residue of FAK in the hippocampus, which is crucial for FAK activation [261] and increased phosphorylation of p130-Cas, a protein associated with FAK in the hippocampus. CB1 receptor-stimulated FAK-autophosphorylation was shown to be upstream of the Src family kinases [261]. These new downstream effectors of CB1 receptors are most likely to play a role in some forms of synaptic plasticity via gene regulation; however, further investigation is required.

There is evidence that perinatal exposure to cannabinoids modifies the maturation of neurotransmitter systems and their related behaviors [213,262,263,264,265]. These effects were caused via the activation of CB1 receptors, which emerge early in the developing brain [212,213,241,265]. Activity in the adult brain of a specific neurotransmitter is the result of a temporally ordered sequence of events that occurs during early CNS development. Dysregulation of this pattern may result in alterations in some of the functions related to this neurotransmitter. For example, a disturbance in the expression of the genes implicated in the synthesis of receptors in a very specific moment of development can result in alterations in some of the activities related to the physiological functions of these receptors. These alterations may also be a consequence of an increase or decrease in their concentrations or a modification in the activity of the CB1 receptor signaling pathways. Administration of cannabinoids, at doses similar to those found in marijuana consumers, was found to modify normal neurotransmitter development, which likely produced neurobehavioral disturbances. Thus, adult animals that are perinatally exposed to cannabinoids exhibit, among other symptoms, long-term alterations in male copulatory behavior [266], open-field activity [267], learning ability [268], stress response [269], pain sensitivity [270], social interaction and sexual motivation [271], drug seeking behavior [272], neuroendocrine disturbances [273] among others (for review, see [262,263,264,268]). Furthermore, most of these neurobehavioral effects were originated by changes in the development of several neurotransmitter systems caused by exposure to cannabinoids and most likely through the activation of CB1 receptors during critical prenatal and early postnatal periods of brain development.

During specific periods of development, CB1 receptors might also be expressed in some subpopulations of glial cells, which play an important role in neural development. Cannabinoids induce arachidonic acid mobilization in cortical glial cells, and this effect was reversed by the selective CB1 receptor antagonist, SR141716A [274], suggesting that the CB1 receptor might play a role in neural-glial signaling in the brain. This may result in AEA being released from neuronal cells, which might act on astrocyte function via the activation of CB1 receptor located in these cells. It has been observed that cannabinoid-induced increase in basal and forskolin-inhibited glucose oxidation and phospholipid synthesis was observed in cortical glial cells [275] or C6 glioma cells [276]. These effects were reversed by pertussis toxin or SR141716A, indicating an involvement of a Gi/Go protein-coupled CB1 receptor. These studies also suggested that sphingomyelin hydrolysis and mitogen-activated protein kinase stimulation were also involved in this metabolic effect [275]. Cannabinoids in hippocampal glial cell cultures induced the expression of *kros-24*, which was reversed by SR141716A [277], suggesting the involvement of CB1 receptors. Similarly, acute administration of Δ^9^-THC (1–10 mg/kg) markedly enhanced the proapoptotic properties of ethanol in the neonatal rat brain. However, Δ^9^-THC did not induce neurodegeneration by itself, although neuronal degeneration became disseminated and severe when Δ^9^-THC was combined with a mildly intoxicating ethanol dose. This Δ^9^-THC and ethanol dose combination resembled the massive neurodegeneration observed when ethanol was administered alone at much higher doses [278]. In addition, Δ^9^-THC and co-administration of a low dose of ethanol enhanced CB1 receptor levels without affecting CB2 receptor levels in the thalamus and dorsal subiculum brain regions. The effect of Δ^9^-THC on neurodegeneration was mimicked by the synthetic cannabinoid WIN 55,212-2 (1–10 mg/kg) and counteracted by the CB1 receptor antagonist SR141716A (0.4 mg/kg). In addition, neonatal CB1 receptor knock-out mice were less susceptible to the neurotoxic effects of a low dose of ethanol. Furthermore, the CB1 receptor antagonist SR141716A ameliorated the neurotoxic effects of ethanol [278].

In our recent studies, we used a higher dose of postnatal ethanol, which induced a massive widespread neurodegeneration in postnatal day 7 mouse brains. In addition to enhanced AEA and associated biosynthetic enzymes, we also found transcriptional activation of the CB1 receptor gene, which results in enhanced levels of CB1 receptor mRNA as well as protein expression in a time-dependent manner in cortical and hippocampal brain regions [111]. Interestingly, we found that postnatal ethanol treatment of mice enhances acetylation of histone (H4) on lysine 8 (H4K8ace) at CB1R exon1, CB1R binding as well as the CB1R agonist-stimulated GTPγS binding in cortical and hippocampal brain regions [110]. Pre-administration of SR141716A or genetic ablation of CB1 receptors (KO) prior to ethanol treatment prevented neurodegeneration [110,111]. Interestingly, the protective effects of CB1R blockade through pharmacological or genetic deletion resulted in normal adult synaptic plasticity and learning and memory including social memory in mice exposed to ethanol at P7. The AEA/CB1R signaling pathway may be directly responsible for the synaptic and memory deficits associated with fetal alcohol spectrum disorders [110,111].

The EC signaling pathway responsible for the specific role during brain development has not been well characterized. The available literature suggests the participation of ERK 1/2 via a mechanism involving the upstream inhibition of Rap1 and B-Raf (for review see [208]). Activation of CB1Rs also prevented the recruitment of new synapses by inhibiting the formation of cAMP [221]. Although the intracellular signaling events involving mitogen-activated protein kinase (MAPK) coupled to the activation of CB1Rs have been shown in embryonic developmental stage [217] and are not known during postnatal development, several studies using cell lines have suggested both up- and downregulation of MAPK in Δ^9^-THC-mediated apoptosis [279,280]. Moreover, cannabis use during brain development also induced a veriety of deficits that are similar to several specific human developmental disorders [281], and may overlap with, those observed in fetal alcohol syndrome [282], which was likely mediated via the activation of CB1Rs.

**Figure 2:**
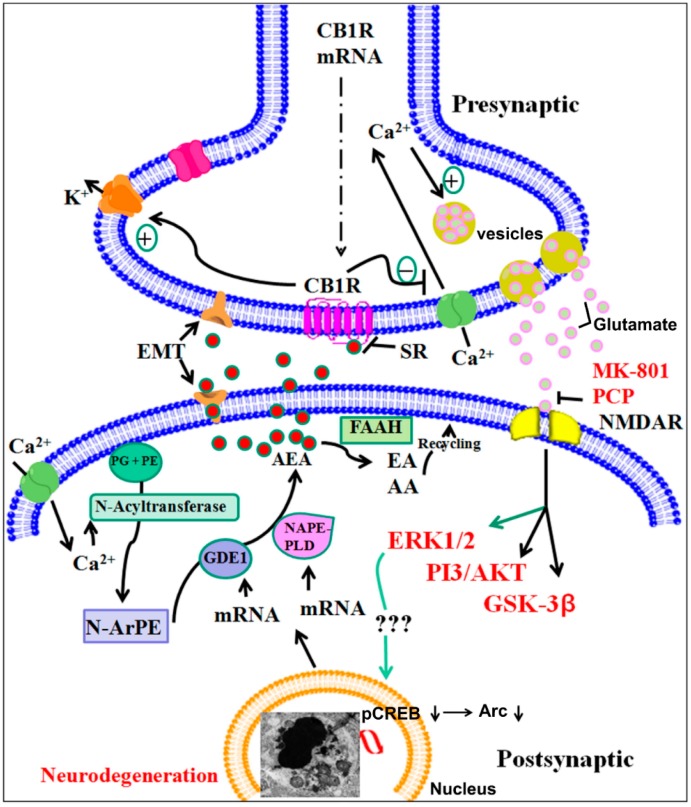
A proposed model suggesting that AEA/CB1R/ERK1/2/pCREB/Arc signaling regulates ethanol-induced neurodegeneration, resulting in neurobehavioral deficits in adult mice. P7 ethanol-enhances AEA levels (postsynaptic neuron) through transcriptional activation of NAPE-PLD and GDE1 enzymes. AEA acting through CB1Rs (AEA tone) (presynaptic neuron) may result in decreased glutamate release, which causes *N* -Methyl-d-aspartate (NMDA) receptor hypofunction, ERK1/2 and cAMP response element-binding protein (CREB) hypophosphorylation deficits in Arc expression and leads to neonatal neurodegeneration (condensed chromatin balls, electron microscopic image). Previous studies have shown that CB1R activation inhibits NMDA receptor function in several experimental models [283,284] and ethanol was shown to inhibit glutamatergic neurotransmission via CB1 receptor activation [285]. These events during postnatal development may disrupt the refinement of neuronal circuits [112,123] and lead to long-lasting deficits in synaptic plasticity and memory in adult animals. The inhibition of CB1Rs (AEA tone) prevents pERK1/2, CREB hypophosphorylation, deficits in Arc expression and neonatal neurodegeneration (Tau and caspase-3 cleavage), which results in normal neurobehavioral function in adult mice. Genetic ablation of the CB1R does not affect NMDA receptor antagonist-induced apoptosis, but does provide protection against ethanol-induced neonatal neurodegeneration, synaptic and memory deficits in adult mice. Thus, the putative AEA/CB1R/pERK1/2/pCREB/Arc signaling mechanism may have a potential regulatory role in neuronal function in the developing brain and may be a valuable therapeutic target for FASD.

Our recent study demonstrated a specific role of CB1 receptor-mediated ERK1/2, cAMP response element-binding protein (CREB) phosphorylation, AKT and activity-regulated cytoskeleton-associated protein (Arc) expression in postnatal ethanol-induced neurodegeneration. P7 ethanol treatment significantly reduced pERK1/2, pAKT, pCREB and Arc protein levels but not total ERK1/2 or AKT or CREB protein levels in the hippocampus and neocortex. Interestingly, ERK1/2, CREB phosphorylation and Arc expression were rescued by SR pre-treatment. However, AKT phosphorylation was not rescued by SR pre-treatment. Similarly, CB1R KO mice, which did not show ethanol-induced neurodegeneration, provided protection against P7 ethanol-induced inhibition of ERK1/2, pCREB phosphorylation and Arc expression but failed to rescue the inhibition of AKT phosphorylation. Thus, P7 ethanol-activated CB1R-induced neurodegeneration was regulated by the AEA/CB1R/pERK1/2/pCREB/Arc cascade but not by the PI3-kinase/AKT pathway in the developing brain [110,111] (Figure 2).

During periods of development when the brain experiences a growth spurt, blockade of the NMDA receptor for a few hours has been found to trigger massive and widespread apoptotic neurodegeneration in the rodent brain [286]. NMDA receptor antagonists were the most effective in inducing apoptosis in the rat forebrain at P7. Thus, during this developmental period, the survival of NMDA receptor-expressing neurons was dependent on the glutamatergic input being regulated within narrow time periods [286]. Endocannabinoids and cannabinoids are well known to affect glutamatergic signaling [287,288], and therefore, ethanol-induced endocannabinoids [111,285] or cannabinoid-induced alterations in glutamate levels [289,290,291] might contribute to neonatal neurodegeneration or lasting behavioral deficits [111,112,123] observed after binge-like ethanol exposure during this specific vulnerable period of brain development. Furthermore, pharmacological blockade or genetic deletion of CB1 receptors removed the endocannabinoid-mediated inhibition of glutamate release by ethanol, resulting in a lack of ethanol-induced neurodegeneration (Figure 2). Thus, CB1 receptors served as good candidate targets for modulating NMDA receptor function in developmental disorders. Interestingly, NMDA receptor antagonists were able to induce apoptotic neurodegeneration in CB1 receptor KO mice [111], further strengthening the mechanism by which postnatal ethanol exerts its deleterious effects in the developing brain (Figure 2). Findings obtained from neonatal rats suggested that ethanol might actually affect CA3 pyramidal neurons via inhibition of postsynaptic amino-3-hydroxy-5-methyl-4-isoxazolepropionic acid receptor (AMPARs), which result in a decrease in glutamatergic release [292]. Moreover, this study was consistent with the study conducted by Twitchell *et al.* in 1997 in which exogenous cannabinoids inhibited glutamatergic release by activating CB1R-mediated inhibition of N-type and P/Q-type calcium channels [283] and might be responsible for the enhanced susceptibility of the immature brain to ethanol neurotoxicity [278] and long-lasting learning and memory deficits [108,109,110,111,112,293].

While the molecular mechanism is still unclear, ethanol treatment during early brain development caused deficits in synaptic function [294], which persisted into adulthood [108,109,110,111,112,293]. It is also possible that ethanol-enhanced AEA-CB1 receptor tone disrupts the refinement of neuronal circuits, leading to persistent synaptic dysfunction. This could explain why some cortical maps [295,296,297,298] and olfacto-hippocampal networks [112,123] are altered in FASD models. Consistent with this notion, pre-administration of CB1 receptor antagonist (SR141716A) completely rescued the P7 ethanol-induced defects in the LTP magnitude of field excitatory postsynaptic currents (fEPSPs), both in the initial induction and maintenance. Interestingly, the genetic deletion of CB1 receptors provided complete protection against P7 ethanol-induced defects in LTP. However, CB1R KO mice exhibited an enhanced LTP magnitude compared to WT or normal C57BL/6J saline-treated mice [109,110,111] as observed in other studies [299,300]. In addition, ethanol treatment at P7 impaired object recognition, spatial and social interaction memory performance and was rescued in mice treated with SR [109,110,111]. P7 ethanol administration showed no significant effects on adult CB1R WT or KO mice exploration times in the object recognition memory (ORM) task. Because deletion of CB1 receptors has been shown to enhance their ability to perform better on a learning task [300], we found enhanced object recognition performance and spatial memory in CB1 receptor KO mice compared to WT mice. Furthermore, CB1 receptor KO mice provided protection against P7 ethanol-induced deficits in object recognition memory performance, spatial memory and social interaction memory as observed in LTP. Therefore, suppression of CB1 receptor function via pharmacological blockade or genetic deletion of CB1 receptors prior to postnatal ethanol exposure prevented synaptic and memory deficits in adult mice. Taken together, these findings emphasize the importance of this mechanism in the understanding and development of therapeutic strategies used to treat FASD.

It is also possible that activation of CB1 receptors during the critical period of brain development can affect both the glutamatergic system and the development of multiple neurotransmitter systems, including the catecholaminergic, serotonergic, GABAergic and opioid systems [213,301,302], resulting in an altered hippocampal circuit and long-term behavioral deficits [303] (Table 2). Activation of CB1 receptors during postnatal development, although less well investigated, may also result in long-term behavioral deficits [304] mediated through NMDA receptor function [111]. However, further research is required to establish the effect of CB1 receptor activation during brain development on the function of multiple neurotransmitter systems, which may cause lasting morphological changes underlying the synaptic and memory deficits.

**Table 2:** Summary of Long-lasting behavioral abnormalities found in animals as consequences of cannabinoid exposure and CB1 receptor activation during brain development.

	Cannabinoid Exposure	References
Learning and memory	Learning deficits in the Morris water maze.	[305]
	Impairment in the inhibitory avoidance test and impaired olfactory short-term memory in the social discrimination task.	[306]
	Poorer performance in homing behavior and impaired active avoidance performance.	[307]
	Disruption of memory retention in the passive avoidance task.	[308]
	Reduced novel object recognition.	[309,310]
	Impaired working memory on Y maze.	[311]
	Reduced social interaction.	[309,310]

## 2. Conclusions

**Figure 3:**
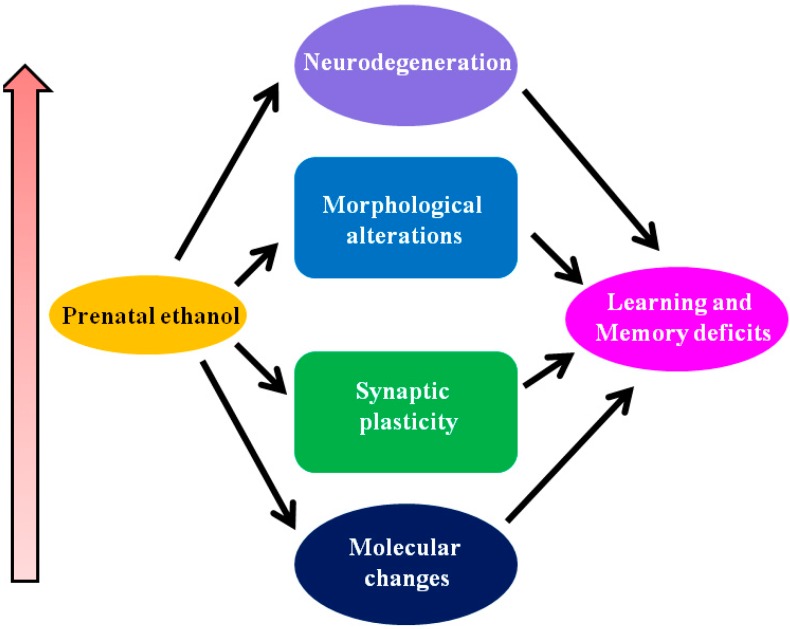
Molecular events leading to loss of learning and memory as found in FASD. Depending on the developmental stage, pattern, concentration and duration of prenatal ethanol exposure can lead to molecular alterations (for example, activity of growth factors, changes in the regulation of gene expression, changes in adhesion, changes in endocannabinoid system) and impaired in neuronal communication and circuit formation. These events may lead to several transient and permanent changes in the hippocampus, including molecular changes, modifications in synaptic plasticity, morphological changes and neuronal loss. These prenatal ethanol-associated changes in the brain can potentially induce deficits in learning-and-memory processes found in the adult animals of FASD.

In summary, several molecular mechanisms are expected to contribute to the damaging effects of prenatal alcohol exposure on the developing fetus. These events including endocannabinoid mechanisms contribute to impaired development and function of neuronal communication and circuit formation. These alcohol-induced deficits result in long-lasting abnormalities in neuronal plasticity and learning and memory and can explain many of the neurobehavioral abnormalities found in FASD (Figure 3). The physiological role of the EC system during early brain development has not yet been fully elucidated. The search for additional functions is underway, and the strategies used to identify them are improving. However, there is not sufficient attention focused in this direction. Modulation of this system using pharmacological and gene knockout approaches support a role for the EC system in learning and memory, emotion and anxiety, reward, eating, nociception and motor systems, among others; however, none of these behavioral responses are critically dependent on the veracity of the EC system, indicating that it functions in a modulatory or facilitatory manner, thus making it a highly attractive target for the development of therapeutic agents to treat FASD.

## Abbreviations

CB1, cannabinoid receptor type 1; EC, endocannabinoid; FAS, fetal alcohol syndrome; FASD, fetal alcohol spectrum disorder; BAC, blood alcohol concentration; ADHD, attention deficit hyperactivity disorder; miRNAs, microRNAs; NCAM, neural cell adhesion molecule; GABAA, gamma-amino butyric acid-A receptor; MAPK, mitogen-activated protein kinase; Δ^9^-THC, delta-9-tetrahydrocannabinol; AEA, arachidonylethanolamide (anandamide); 2-AG, 2-arachidonylglycerol; NADA, *N*-arachidonyl-dopamine; VR1, vanilloid receptor type 1; NAPE-PLD, *N*-acylphosphatidylethanolamine-specific phospholipase D; GDE1, glycerophosphodiesterase; ABHD4, abhydrolase domain 4; PTPN22, phosphatase; AMT, AEA membrane transporter; FAAH, fatty acid amide hydrolase; COX, cyclooxygenase; LOX, lipoxygenase; MAGL, monoacylglycerol lipase; PGH2, prostaglandin H2; BDNF, brain-derived neurotrophic factor; DAGL, diacylglycerollipase; FAK, focal adhesion kinase; AMPAR, α-amino-3-hydroxy-5-methyl-4-isoxazolepropionic acid receptor; fEPSCs, field excitatory postsynaptic currents; GPCR, G protein coupled receptor; NAT, *N*-acyltransferase; N-ArPE, *N*-arachidonyl phosphatidylethanolamine; NMDA, *N*-methyl-d-aspartate.
